# Action Sport Cameras as an Instrument to Perform a 3D Underwater Motion Analysis

**DOI:** 10.1371/journal.pone.0160490

**Published:** 2016-08-11

**Authors:** Gustavo R. D. Bernardina, Pietro Cerveri, Ricardo M. L. Barros, João C. B. Marins, Amanda P. Silvatti

**Affiliations:** 1 Department of Physical Education, Universidade Federal de Viçosa, Minas Gerais, Brasil; 2 Department of Electronics, Information and Bioengineering, Politecnico di Milano, Milan, Itália; 3 Faculty of Physical Education, Universidade Estadual de Campinas, São Paulo, Brasil; Coastal Carolina University, UNITED STATES

## Abstract

Action sport cameras (ASC) are currently adopted mainly for entertainment purposes but their uninterrupted technical improvements, in correspondence of cost decreases, are going to disclose them for three-dimensional (3D) motion analysis in sport gesture study and athletic performance evaluation quantitatively. Extending this technology to sport analysis however still requires a methodologic step-forward to making ASC a metric system, encompassing ad-hoc camera setup, image processing, feature tracking, calibration and 3D reconstruction. Despite traditional laboratory analysis, such requirements become an issue when coping with both indoor and outdoor motion acquisitions of athletes. In swimming analysis for example, the camera setup and the calibration protocol are particularly demanding since land and underwater cameras are mandatory. In particular, the underwater camera calibration can be an issue affecting the reconstruction accuracy. In this paper, the aim is to evaluate the feasibility of ASC for 3D underwater analysis by focusing on camera setup and data acquisition protocols. Two GoPro Hero3+ Black (frequency: 60Hz; image resolutions: 1280×720/1920×1080 pixels) were located underwater into a swimming pool, surveying a working volume of about 6m^3^. A two-step custom calibration procedure, consisting in the acquisition of one static triad and one moving wand, carrying nine and one spherical passive markers, respectively, was implemented. After assessing camera parameters, a rigid bar, carrying two markers at known distance, was acquired in several positions within the working volume. The average error upon the reconstructed inter-marker distances was less than 2.5mm (1280×720) and 1.5mm (1920×1080). The results of this study demonstrate that the calibration of underwater ASC is feasible enabling quantitative kinematic measurements with accuracy comparable to traditional motion capture systems.

## Introduction

Motion capture systems are traditionally adopted to reconstruct the movements of animals and humans in different applications such as biomechanics [1–2], sport gesture analysis [3–7], rehabilitation [8–9] and clinics [10–11]. However, optoelectronics and electromagnetic devices, mainly devoted to laboratory analysis, feature high costs and are not designed for both outdoor and underwater usage. Qualisys company distributes a video-based commercial system [12], specifically designed for underwater measurements using devoted illumination to enhance image quality. However, cameras still demand cables and the system is very expensive.

An alternative video-based technology is represented by action sport cameras (ASC), which are currently used mainly for recreational purposes. Their uninterrupted technical improvements, in terms of image resolution and capture frequency, in correspondence of a cost decrease, are enabling them to sport gesture study and athletic performance evaluation [13–17]. Recent works in the literature described the application of ASC for two-dimensional (2D) analysis [18–21]. Extending this technology for three-dimensional (3D) sport analysis using multiple cameras, however, still requires a methodologic step-forward to making ASC a metric system, encompassing ad-hoc camera setup, synchronization of the acquisitions, and devoted calibration protocols.

In swimming motion analysis for example, the camera setup is particularly demanding as the athletic gesture develops both in air and underwater concurrently. ASC manufacturers partially addressed this issue by developing different accessories, especially designed for underwater usage, as waterproof housings and support with suction cups to secure the cameras to the wall of the swimming pool. From an operational point of view, camera calibration represents a bottleneck to the development of video-based underwater motion analysis systems because of two main issues. First, the 3D reconstruction of the complete athletic gesture requires the calibration of both air and underwater cameras and a coordinate system registration in between them. At present time however, there are no standardized protocols available. Second, underwater calibration can require specific solutions addressing water disturbance of the image quality to ensure high reconstruction accuracy.

In order to achieve high accurate 3D underwater movement analysis, our group already addressed some critical points related mainly to underwater camera calibration. Using industrial cameras, we showed that the accuracy results of the wand-based and 2D plate-based calibration methods were less associated to the testing tool position in the working volume and provided better accuracy than the graduated rod-based calibration with nonlinear DLT [22]. The main advantage of using wand-based calibration was the equalization of the reconstruction error across the working volume, ensured by the bundle adjustment of the camera parameters. In contrast, 2D plate-based calibration led to an unregistered camera network as each camera was calibrated separately. However, 2D plate-based calibration was less sensitive to water quality than wand-based. In the [23], we applied wand-based underwater calibration to reconstruct with high accuracy the hand trajectory of four swimmers during front-crawl, breaststroke and butterfly styles.

In this paper, the underwater wand-based calibration procedure was applied to the ASC. Experimentally, two cameras were submerged into a swimming pool and located steady at the corners of a working volume of about 6m^3^. The measurement protocol encompassed calibration and testing acquisitions to compute camera parameters and evaluate the reconstruction accuracy, respectively, with two different image resolutions. The effect of a set of calibration data acquisition strategies, on the 3D accuracy, was investigated.

## Materials and Methods

### Instruments

**Fig 1:**
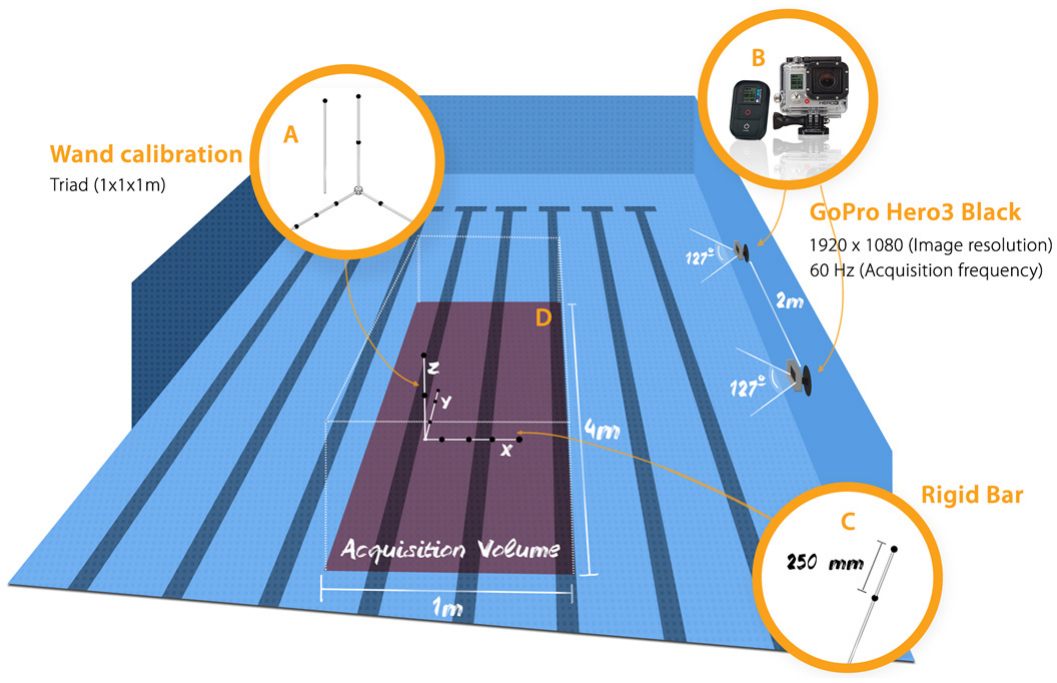
A) Calibration tools. B) Action sport cameras (GoPro, Hero3+, Black). C) Rigid bar used to 3D reconstruction accuracy evaluation. D) Camera position and acquisition volume.

The data acquisition was performed in a swimming pool. Two action sport cameras with waterproof housings (GoPro, Hero3+, Black Edition^®^ / USA), were fixed on the wall of the swimming pool (see Fig 1). The view angle and acquisition frequency were 127° and 60Hz, respectively. Two different image resolutions were investigated, namely 1280×720 (LOWRES) and 1920×1080 (HIGHRES) pixels. In order to synchronize the cameras, we used the Wi-Fi remote GoPro control (see Fig 1B). After acquisition, the videos were converted to AVI movie format in the GoPro studio software.

### Camera calibration procedure

**Fig 2:**
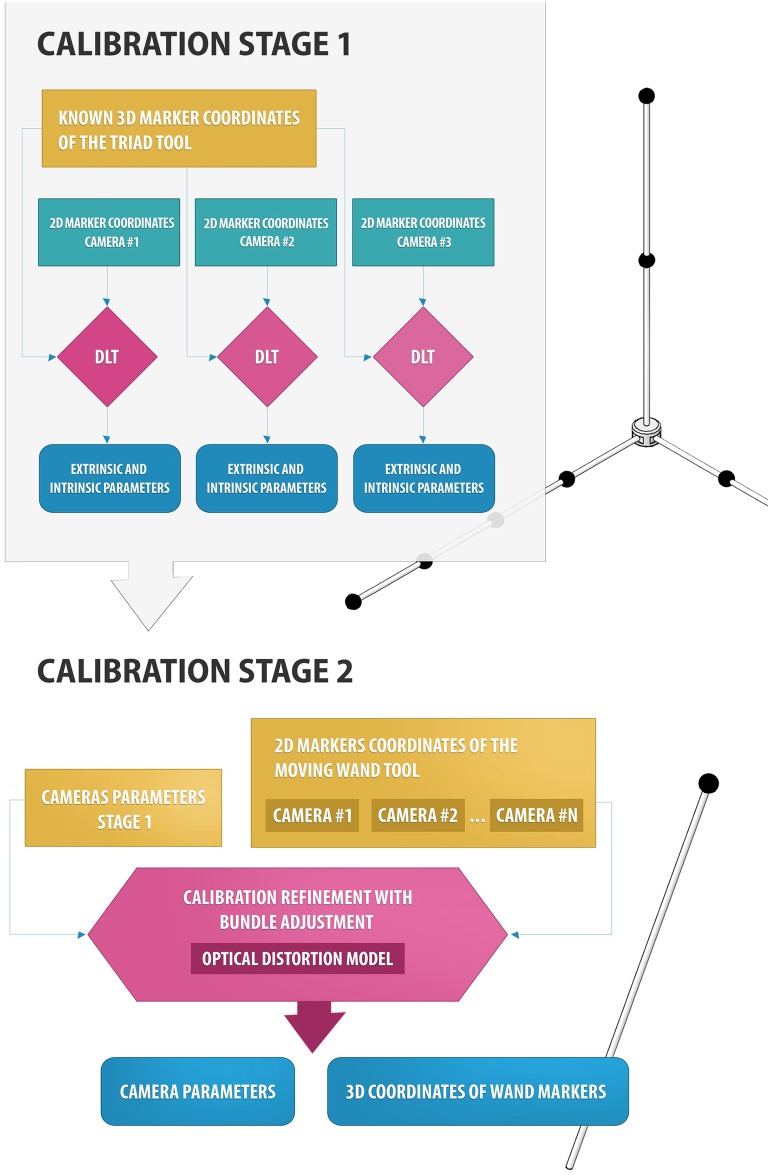
Schematic workflow of the two-stage camera calibration for a generic number of cameras.

The wand-based calibration method consists in the acquisition of one static (a triad structure) and one moving (a wand structure) tool, carrying nine and one spherical passive markers, respectively. One waterproof orthogonal triad structure (1×1×1m) was built by a computer numerical control machine (CNC) screwing onto it nine spherical black markers (∅: 35 mm) in known positions (10μm accuracy). The triad was located at the floor of the swimming pool, in the center of the working volume (1×4×1.5m^3^) and acquired for 5 seconds (see Fig 1A). 2D marker segmentation in videos and centroid computation were performed using “Dvideo” software [24]. 2D data of triad markers were used to assess the initial intrinsic and extrinsic parameters of the cameras, using DLT method disregarding optical distortions [22], and define the origin and orientation of the working volume. In order to refine the camera parameters, also ensuring nonlinear optical distortion correction [25], a wand, carrying one spherical marker located at its extremity, was moved in the working volume, during about 20 seconds. “Dvideo” software was used again to track the marker in the image sequence of the two cameras. Four hundred useful video frames were used into a bundle adjustment nonlinear optimization, using control points with both known (triad markers) and unknown (wand marker) 3D coordinates [26]. The bundle adjustment iteratively estimates the parameters of all the cameras along with the unknown 3D marker coordinates by minimizing the 2D projection error (measured vs predicted by the camera model) on the images. The optical distortion was taken into account by adding one radial parameter into the camera model (Fig 2).

### Calibration quality

The calibration quality was evaluated by the accuracy of 3D reconstruction in both image resolutions (HIGHRES and LOWRES). We used five acquisitions of the rigid bar, carrying two markers at known distance (*d*_*n*_: 250 mm), moved within the working volume during 15 seconds (Fig 1C). The wand was manufactured by CNC machine ensuring a marker positioning accuracy of about 10μm. In order to describe the 3D accuracy of ASC the following quantities were calculated for each trial: a) mean value of the marker distance; b) the standard deviation of the distance distribution; c) the mean absolute error (difference between the nominal and the measured marker distances); d) the percentage accuracy (the ratio between the absolute accuracy and the maximum diagonal of the working volume) [27]. The error distributions for HIGHRES and LOWRES were compared using a non-parametric test (Wilcoxon rank sum) with a significance of 1%. The calibration quality was also evaluated in laboratory and were compared to the underwater condition, using same camera setup (two cameras, HIGHRES and LOWRES, 60Hz, camera position) and calibration protocol (triad and wand tools).

### Calibration dependability

**Fig 3:**
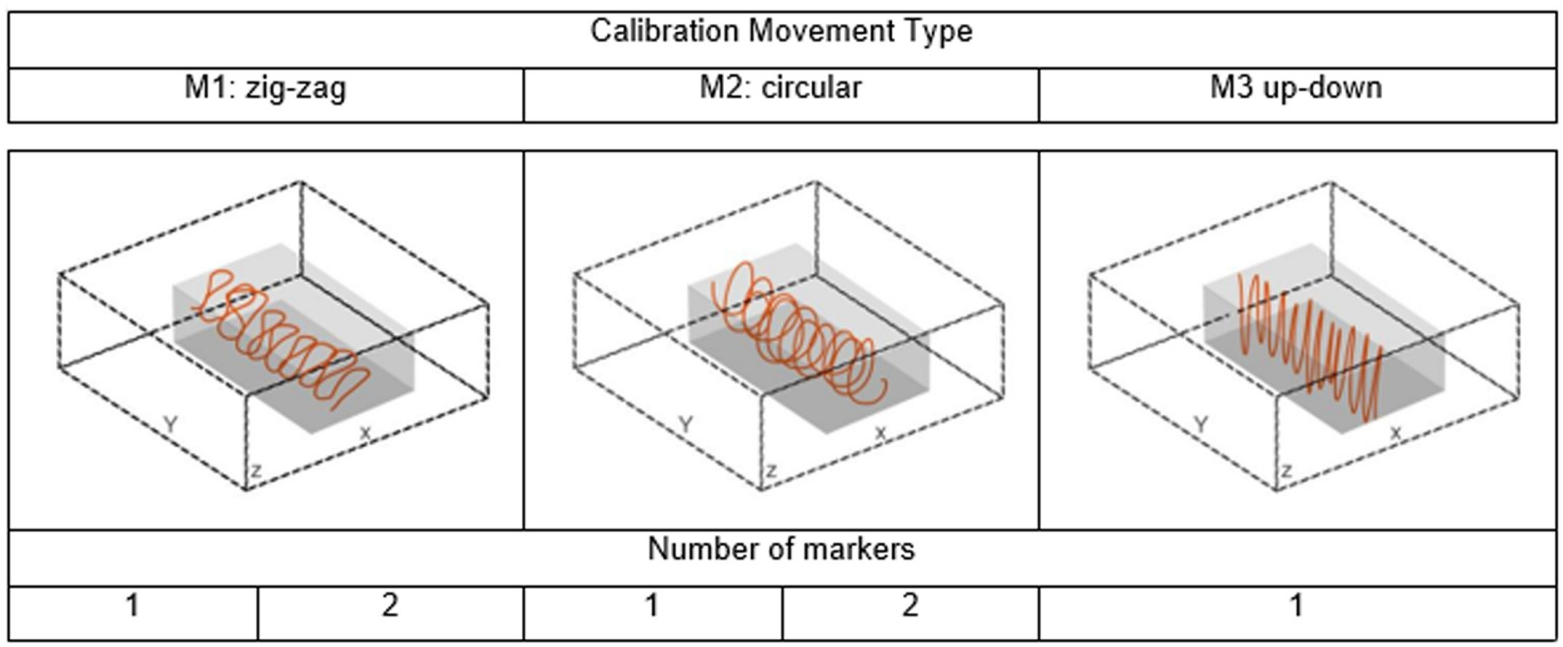
Three different calibration wand movements to evaluate the calibration dependability.

Since the calibration quality can be affected by the performed wand acquisition movement [26], we evaluated the camera dependability testing three different acquisitions, namely zig-zag (M1), circular (M2) and up and down (M3). For this test, we used the high image resolution. As far as M1 is concerned, the operator was instructed to well cover all the camera field of view. In the M2 wand movement, the operator was instructed to perform circular movements within the working volume. In M3, the operator was instructed to move up-down the wand within the working volume. In addition, we evaluated whether to add an inter-marker distance constraint into the bundle adjustment (two-marker in the wand tool) could result in an improvement of the 3D reconstruction accuracy. Operatively, five different calibration tests (see Fig 3), explicitly M1-1 (one marker with zig-zig movement), M2-1 (one marker with circular movement), M3-1 (one marker with up-down), M1-2 (two markers with zig-zig movement) and M2-2 (two markers with circular movement), were performed. The quality of all the five calibrations was assessed again by reconstructing the distance between the markers of the test rigid bar moved within the working volume during about 20s.

In order to analyze how the wand movement type and the number of markers affected the calibration dependability, we calculated the minimum, mean and maximum value of the distance between the markers, the standard deviation and the mean absolute error, in all the five acquisition protocols (M1-1, M2-1, M3-1, M1-2 and M2-2). The five distance error distributions were statistically analyzed by using a non-parametric test (Kruskal-Wallis) with a *post-hoc* (Tukey: *p-value*<0.05) (Matlab^®^ 2012).

## Results

### Calibration quality

In the five repeated calibrations, the reconstruction error was below 2.6mm for both image resolutions. In Table 1, we listed mean of the distance between the markers, standard deviation and mean absolute errors (bias) of the five trials of the dynamic rigid bar test (2 markers).

**Table 1:** Results of the 5 trials of dynamic rigid bar test (HIGHRES and LOWRES). Nominal distance *d*_*n*_ between the two markers: 250mm.

Trial	Mean ± SD (mm)		Mean Absolute Error (mm)		Error related to volume size	
	HIGHRES	LOWRES	HIGHRES	LOWRES	HIGHRES	LOWRES
1	249.95±1.81	248.0±1.70	1.36	2.22	1:3000	1:5200
2	250.27±1.67	247.8±1.30	1.29	2.28	1:3000	1:5300
3	249.91±1.55	247.86±1.78	1.22	2.37	1:3000	1:5500
4	250.11±1.71	247.51±1.78	1.35	2.63	1:3000	1:6100
5	250.04±1.48	247.60±1.65	1.19	2.55	1:3000	1:6000
Land	248.53±1.08	248.56±1.40	1.56	1.67	1:3600	1:3800

**Fig 4:**
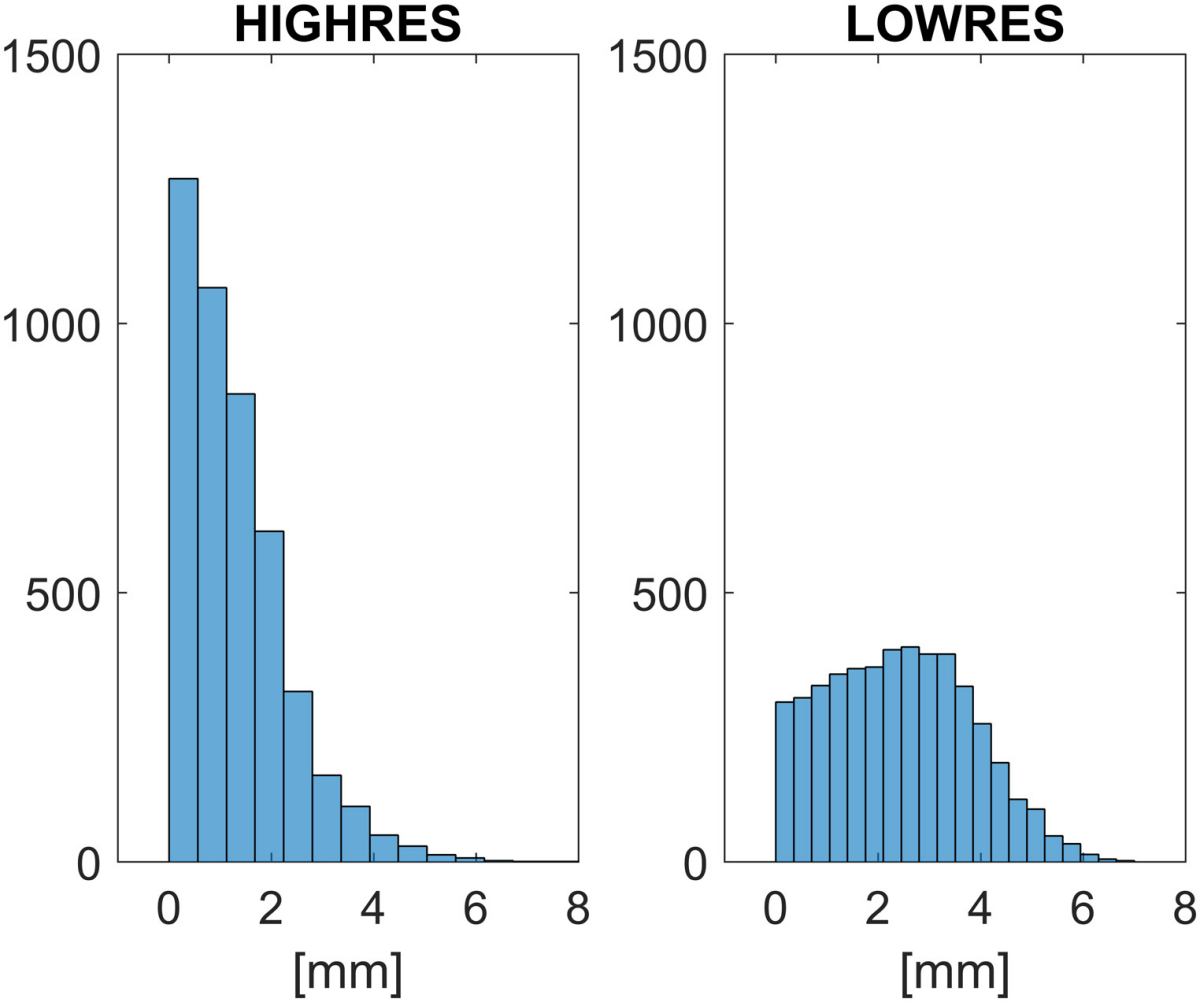
The histograms of the residual error distribution (cumulated over the five trials) for HIGHRES (1920–1080) and LOWRES (1280–720). The average values were 1.28 and 2.41mm, respectively.

As expected, the bias of HIGHRES was lower (1.28mm) than that of LOWRES (2.41mm). The two error distributions were statistically (*p-value*<0.0001) different (see Fig 4). For comparison, the 3D reconstruction in laboratory using HIGHRES and LOWRES led to a mean absolute error of 1.56mm and 1.67mm, respectively.

### Calibration dependability

There was a significant difference (*p*<0.001) among the five different calibrations (Table 2). Since the movement M1 spread more the working volume, we found the best accuracy results in M1-1 (1.17 mm) and M1-2 (1.28 mm).

**Table 2:** Minimum, mean and maximum inter-marker distance, averaged across 5 trials and the corresponding mean absolute error (*d*_*n*_: 250mm). M1-1 (one marker with zig-zig movement); M2-1 (one marker with circular movement); M3-1 (one marker with up-down); M1-2 (two markers with zig-zig movement); M2-2 (two markers with circular movement). The post-hoc comparison results were reported (*p<0.05).

Calibration	Distance (mm)			Mean Absolute Error (mm)	Post-hoc comparison	*p-value*
	Minimum	Mean	Maximum			
**M1-1**	249.31	249.43	249.55	1.17	M1-2	0.08
					M2-1	0.000*
					M2-2	0.01*
					M3-1	0.000*
**M1-2**	249.07	249.15	249.23	1.28	M2-1	0.000*
					M2-2	0.06
					M3-1	0.000*
**M2-1**	248.57	248.60	248.67	1.80	M2-2	0.000*
					M3-1	0.000*
**M2-2**	248.89	248.95	249.08	1.47	M3-1	0.000*
**M3-1**	247.92	248.13	248.29	2.63		

No significant difference was found when we compared M1 movement using or not the distance constraints (Table 2). When the movement did not spread systematically the working volume (M2), the usage of the distance constraint in the bundle calibration improved significantly the accuracy of results (M2-1 = 1.80mm, M2-2 = 1.47mm, *p*<0.001). M3-1 acquisition protocol (non-systematic movement and one marker wand) yielded the worst reconstruction error (2.63mm). As noticed, when considering M1-2 and M2-2 (different movements but with bar length constraint), no statistical difference was found.

## Discussion

Performing 3D kinematic analysis in sports, as in the case of swimming, requires high reconstruction accuracy. The usage of nonlinear camera calibration was reported to improve the accuracy results found in laboratory conditions [25, 28, 29]. Reconstruction errors, ranging from 0.58 to about 1mm, were obtained when using optoelectronic systems and industrial cameras [15, 30, 23]. The reconstruction accuracy of ASC (wide-angle lenses—GoPro) was evaluated out of water conditions obtaining 10mm error in correspondence of a linear camera model [31]. In the same paper, the authors reported that the accuracy increased by five times (2mm) when adopting a nonlinear camera model. This last result is in agreement with our GoPro laboratory test (cfr. Table 1).

Underwater 3D analysis, based on cumbersome calibration structure and linear camera models, using traditional video-based systems, provided a reconstruction error higher than 5mm [32–35]. In [22], it was shown that underwater camera calibration using industrial cameras and nonlinear camera model, improves on average the reconstruction accuracy up to 1mm across a working volume of about 7m^3^. This result was comparable with the values (2mm at 10m distance) reported by commercial systems devoted to 3D underwater analysis [12].

In the light of such prior results, the present study evaluated the reconstruction accuracy of underwater ASC calibrated using the wand method with a nonlinear camera model encompassing optical distortions [22]. We found that the average error upon the reconstructed inter-marker distances was less than 1.5mm (HIGHRES underwater and Land) on average across the whole working volume of about 6m^3^, comparable with the values reported in our previous work [22], with reconstruction errors reported in [36] using GoPro cameras and in [12] using the Oqus-Underwater system.

As far as the calibration dependability is concerned, the wand calibration movement, as expected, affected the reconstruction accuracy results. As shown (cfr. Table 2), spreading the wand systematically across the whole working volume (M1), led to the best accuracy results. The M3 protocol, featuring up-down wand movements without any systematic control, led in contrast to the poorest results. Circular movement (M2), while not systematically covering the entire working volume, ensured accuracy results less than 2mm, nonetheless worse than the results obtained with M1. The analysis of the calibration dependability related to the number of markers upon the calibration wand showed that one marker is sufficient when it is acquired well spread within the whole working volume. We point out that the use of two markers (distance constraint) can improve the reconstruction accuracy making the result less dependent on the wand movement performed by the operator (see Table 2: M1-2 vs M2-2). Based on these considerations, we can argue that the wand-based calibration makes ASC suitable competitive to industrial cameras for underwater motion analysis.

**Fig 5:**
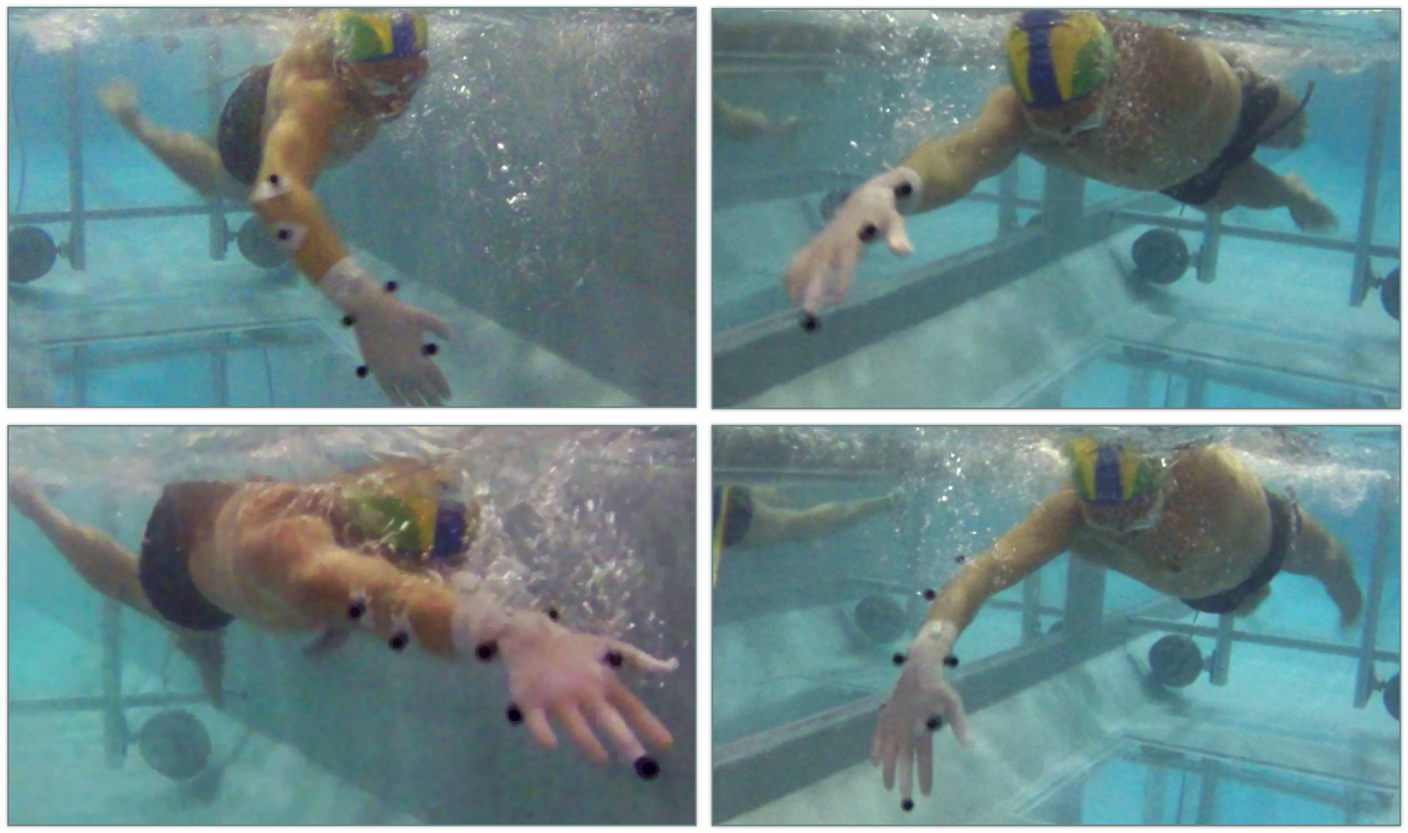
Two instants of the front-crawl swim cycle surveyed by two cameras. The swimmer is equipped with surface markers attached to the right arm. This real condition elucidates how poor image contrast and water disturbance can complicate the automatic marker detection on the image and tracking analysis, affecting the accuracy of the 3D kinematic analysis.

Some limits of the present study must be however discussed. First, we performed an evaluation of the potentiality of ASC in terms of calibration setup and reconstruction accuracy, disregarding the environmental issues relative to underwater conditions. For instance, the image contrast, which can be affected by the illumination of swimming pool, and the water disturbance, which is directly related to the speed of the swimmer, deteriorate the marker detection quality on the images (see Fig 5). Such environmental issues demand therefore specific testing to evaluate their effects on the 3D reconstruction accuracy. We plan to perform a systematic analysis of this effect in future works.

Second, the issue relative to the 3D kinematic analysis underwater is concerning the marker protocol utilized to compute the absolute and angular kinematics. While increasing the number of markers ensures a better body segment definition, marker labeling and tracking procedures complicate and the swimmer performance would be greater affected by water drag increase [37]. In order to avoid it, the use of crosses or circles drawn on the swimmer skin was proposed [38]. Alternatively, a complete markerless approach could be applied as described in [39–40]. However, the segmentation of the complete swimmer silhouette on the images demand a complex network of camera with an enhanced underwater illumination to increase swimmer-to-water contrast [40].

Third, we did not perform an extensive evaluation of the role of the GoPro acquisition setup (image resolution, acquisition frequency and view angle of the camera). In this work, we aimed at studying the calibration accuracy for a typical size of the working volume ensuring at least one complete front crawl cycle. Arranging a 6m working volume wide and camera-to-working volume distance of about 3m required a camera view angle of about 130°. The only option from GoPro setup to cope with such demands was “medium”, featuring a view angle of 127°. With this camera setup we had available 24Hz, 30Hz, 48Hz and 60Hz frequencies. In order to cope with typical swimmer speed we choose 60Hz. A more systematic comparison among different setup, allowed by GoPro (resolution, view angle and acquisition frequency), will be the subject of future evaluations.

Fourth, we did not consider wearable technology for benchmark comparison. Especially, inertial-magnetic measurements units (IMMUs) have been recently proposed in the literature for underwater kinematic analysis [41]. While being in principle plug & play as they are wireless, these sensors can affect the swimmer performance due to the drag effect, which is augmented as the swimmer speed increases. It has to be pointed out that the drag causes for instance vibrations of the sensors, affecting the quality of the kinematic measure. In addition, to ensure underwater wireless data transmission, high capacity batteries are mandatory to cope with power consumption, increasing the size of the wearable devices.

## Conclusions

This article was conceived to mainly demonstrate the feasibility of the quantitative 3D measurements underwater using action sport cameras. We showed that, by endowing action sport cameras with an opportune calibration methodology (handy tools and bundle adjustment), they can be made an accurate metric system. Compared to optoelectronic devices, especially designed for 3D motion analysis, this technology features low cost, reduced size, high portability, wireless facility and waterproof housings. Swimming, underwater gait, water aerobics, water polo are relevant potential applications for such an emerging technology.

## Supporting Information

S1 Supporting Information **S1.1_Calibration Quality > S1.1.1_Underwater > S1.1.1.1_Highres > S1.1.1.1.1_Calibration > File A. This is the BUNDLE_CameraParameters.** This is the camera calibration parameters. **File B. This is the Triad.** This is the 2D coordinates of the waterproof orthogonal triad structure carrying nine spherical black markers. **File C. This is the WandCalibration.** This is the 2D coordinates of the wand structure carrying one spherical black marker. **S1.1.1.1.2_Accuracy > File D. This is the Trial_1.** This is the 2D coordinates of the first rigid bar test acquisition, carrying two markers at known distance. **File E. This is the Trial_2.** This is the 2D coordinates of the second rigid bar test acquisition, carrying two markers at known distance. **File F. This is the Trial_3.** This is the 2D coordinates of the third rigid bar test acquisition, carrying two markers at known distance. **File G. This is the Trial_4.** This is the 2D coordinates of the fourth rigid bar test acquisition, carrying two markers at known distance. **File H. This is the Trial_5.** This is the 2D coordinates of the fifth rigid bar test acquisition, carrying two markers at known distance. **File I. This is the MotionSequence3D_Trial_1.** This is the 3D coordinates of the first rigid bar test acquisition. **File J. This is the MotionSequence3D_Trial_2**. This is the 3D coordinates of the second rigid bar test acquisition. **File K. This is the MotionSequence3D_Trial_3**. This is the 3D coordinates of the third rigid bar test acquisition. **File L. This is the MotionSequence3D_Trial_4.** This is the 3D coordinates of the fourth rigid bar test acquisition. **File M. This is the MotionSequence3D_Trial_5.** This is the 3D coordinates of the fifth rigid bar test acquisition. **S1.1.1.2_Lowres > S1.1.1.2.1_Calibration > File N. This is the BUNDLE_CameraParameters.** This is the camera calibration parameters. **File O. This is the Triad.** This is the 2D coordinates of the waterproof orthogonal triad structure carrying nine spherical black markers. **File P. This is the WandCalibration.** This is the 2D coordinates of the wand structure carrying one spherical black marker. **S1.1.1.2.2_Accuracy > File Q. This is the Trial_1.** This is the 2D coordinates of the first rigid bar test acquisition, carrying two markers at known distance. **File R. This is the Trial_2.** This is the 2D coordinates of the second rigid bar test acquisition, carrying two markers at known distance. **File S. This is the Trial_3.** This is the 2D coordinates of the third rigid bar test acquisition, carrying two markers at known distance. **File T. This is the Trial_4.** This is the 2D coordinates of the fourth rigid bar test acquisition, carrying two markers at known distance. **File U. This is the Trial_5.** This is the 2D coordinates of the fifth rigid bar test acquisition, carrying two markers at known distance. **File V. This is the MotionSequence3D_Trial_1.** This is the 3D coordinates of the first rigid bar test acquisition. **File W. This is the MotionSequence3D_Trial_2.** This is the 3D coordinates of the second rigid bar test acquisition. **File X. This is the MotionSequence3D_Trial_3.** This is the 3D coordinates of the third rigid bar test acquisition. **File Y. This is the MotionSequence3D_Trial_4.** This is the 3D coordinates of the fourth rigid bar test acquisition. **File Z. This is the MotionSequence3D_Trial_5.** This is the 3D coordinates of the fifth rigid bar test acquisition. **S1.1.2_Land > S1.1.2.1_Highres > S1.1.2.1.1_Calibration > File AA. This is the BUNDLE_CameraParameters.** This is the camera calibration parameters. **File AB. This is the Triad.** This is the 2D coordinates of the waterproof orthogonal triad structure carrying nine spherical black markers. **File AC. This is the WandCalibration.** This is the 2D coordinates of the wand structure carrying one spherical black marker. **S1.1.2.1.2_Accuracy > File AD. This is the Trial_Land.** This is the 2D coordinates of the first rigid bar test acquisition, carrying two markers at known distance. **File AE. This is the MotionSequence3D_Land.** This is the 3D coordinates of the first rigid bar test acquisition, carrying two markers at known distance. **S1.1.2.2_Lowres > S1.1.2.2.1_Calibration File AF. This is the BUNDLE_CameraParameters.** This is the camera calibration parameters. **File AG. This is the Triad.** This is the 2D coordinates of the waterproof orthogonal triad structure carrying nine spherical black markers. **File AH. This is the WandCalibration.** This is the 2D coordinates of the wand structure carrying one spherical black marker. **S1.1.2.2.2_Accuracy > File AI. This is the Trial_Land.** This is the 2D coordinates of the first rigid bar test acquisitions, carrying two markers at known distance. **File AJ. This is the MotionSequence3D_Land.** This is the 3D coordinates of the first rigid bar test acquisitions. **S1.2_Calibration Dependability > S1.2.1_Calibration > File AK. This is the BUNDLE_CameraParameters_Cal_Mov1_1p.** This is the camera calibration parameters of the first movement (M1) with one marker. **File AL. This is the BUNDLE_CameraParameters_Cal_Mov1_2p.** This is the camera calibration parameters of the first movement (M1) with two markers. **File AM. This is the BUNDLE_CameraParameters_Cal_Mov2_1p.** This is the camera calibration parameters of the second movement (M2) with one marker. **File AN. This is the BUNDLE_CameraParameters_Cal_Mov2_2p.** This is the camera calibration parameters of the second movement (M2) with two markers. **File AO. This is the BUNDLE_CameraParameters_Cal_Mov3_1p.** This is the camera calibration parameters of the third movement (M3) with one marker. **File AP. This is the Triad.** This is the 2D coordinates of the waterproof orthogonal triad structure carrying nine spherical black markers. **File AQ. This is the Cal_Mov1_1p.** This is the 2D coordinates of the wand structure carrying one spherical black marker using the first movement (M1). **File AR. This is the Cal_Mov1_2p.** This is the 2D coordinates of the wand structure carrying two spherical black markers using the first movement (M1). **File AS. This is the Cal_Mov2_1p.** This is the 2D coordinates of the wand structure carrying one spherical black marker using the second movement (M2). **File AT. This is the Cal_Mov2_2p.** This is the 2D coordinates of the wand structure carrying two spherical black markers using the second movement (M2). **File AU. This is the Cal_Mov3_1p.** This is the 2D coordinates of the wand structure carrying one spherical black marker using the third movement (M3). **S1.2.2_Accuracy > File AV. This is the Trial_1.** This is the 2D coordinates of the first rigid bar test acquisition, carrying two markers at known distance. **File AW. This is the Trial_2.** This is the 2D coordinates of the second rigid bar test acquisition, carrying two markers at known distance. **File AX. This is the Trial_3.** This is the 2D coordinates of the third rigid bar test acquisition, carrying two markers at known distance. **File AY. This is the Trial_4.** This is the 2D coordinates of the fourth rigid bar test acquisition, carrying two markers at known distance. **File AZ. This is the Trial_5.** This is the 2D coordinates of the fifth rigid bar test acquisition, carrying two markers at known distance. **S1.2.2.1_Mov1_1p > File BA. This is the MotionSequence3D_Trial_1.** This is the 3D coordinates of the first rigid bar test acquisition. **File BB. This is the MotionSequence3D_Trial_2.** This is the 3D coordinates of the second rigid bar test acquisition. **File BC. This is the MotionSequence3D_Trial_3.** This is the 3D coordinates of the third rigid bar test acquisition. **File BD. This is the MotionSequence3D_Trial_4.** This is the 3D coordinates of the fourth rigid bar test acquisition. **File BE. This is the MotionSequence3D_Trial_5.** This is the 3D coordinates of the fifth rigid bar test acquisition. **S1.2.2.2_Mov1_2p > File BF. This is the MotionSequence3D_Trial_1.** This is the 3D coordinates of the first rigid bar test acquisition. **File BG. This is the MotionSequence3D_Trial_2.** This is the 3D coordinate of the second rigid bar test acquisition. **File BH. This is the MotionSequence3D_Trial_3.** This is the 3D coordinates of the third rigid bar test acquisition. **File BI. This is the MotionSequence3D_Trial_4.** This is the 3D coordinates of the fourth rigid bar test acquisition. **File BJ. This is the MotionSequence3D_Trial_5.** This is the 3D coordinates of the fifth rigid bar test acquisition. **S1.2.2.3_Mov2_1p > File BK. This is the MotionSequence3D_Trial_1.** This is the 3D coordinates of the first rigid bar test acquisition. **File BL. This is the MotionSequence3D_Trial_2.** This is the 3D coordinates of the second rigid bar test acquisition. **File BM. This is the MotionSequence3D_Trial_3.** This is the 3D coordinates of the third rigid bar test acquisition. **File BN. This is the MotionSequence3D_Trial_4.** This is the 3D coordinates of the fourth rigid bar test acquisition. **File BO. This is the MotionSequence3D_Trial_5.** This is the 3D coordinates of the fifth rigid bar test acquisition. **S1.2.2.4_Mov2_2p > File BP. This is the MotionSequence3D_Trial_1.** This is the 3D coordinates of the first rigid bar test acquisition. **File BQ. This is the MotionSequence3D_Trial_2.** This is the 3D coordinates of the second rigid bar test acquisition. **File BR. This is the MotionSequence3D_Trial_3.** This is the 3D coordinates of the third rigid bar test acquisition. **File BS. This is the MotionSequence3D_Trial_4.** This is the 3D coordinate of the fourth rigid bar test acquisition. **File BT. This is the MotionSequence3D_Trial_5.** This is the 3D coordinates of the fifth rigid bar test acquisition. **S1.2.2.5_Mov3_1p > File BU. This is the MotionSequence3D_Trial_1.** This is the 3D coordinates of the first rigid bar test acquisition. **File BV. This is the MotionSequence3D_Trial_2.** This is the 3D coordinates of the second rigid bar test acquisition. **File BW. This is the MotionSequence3D_Trial_3.** This is the 3D coordinates of the third rigid bar test acquisition. **File BX. This is the MotionSequence3D_Trial_4.** This is the 3D coordinates of the fourth rigid bar test acquisition. **File BY. This is the MotionSequence3D_Trial_5.** This is the 3D coordinates of the fifth rigid bar test acquisition.
(ZIP)
